# BMI, weight change, appetite reduction and cognitive impairment of elderly patients with diabetes

**DOI:** 10.1038/s41598-024-65005-4

**Published:** 2024-06-18

**Authors:** Gongwu Ding, Meng Lu, Jinlei Li

**Affiliations:** 1 Peking Union Medical College, No.9 Dongdansantiao, Beijing, 100730 China; 2 No. 8 Shijiazhuang Hospital, Shijiazhuang, 050080 China

**Keywords:** BMI, Weight change, Cognitive impairment, Diabetes, Endocrine system and metabolic diseases, Neurological disorders

## Abstract

Body weight is related to both diabetes and cognitive impairment; however, the associations between body mass index (BMI) and cognitive impairment have been reported less frequently among diabetes patients. A total of 1355 patients with type 2 diabetes aged ≥ 60 years were included in this study. The Montreal Cognitive Assessment (MoCA) was administered to assess participants’ cognitive status. We collected self-reported body weight, weight loss and appetite loss data using questionnaires. Associations between body weight status (in childhood, midlife age, and late life), weight loss, appetite changes and cognitive impairment were explored using logistic regression. Among the participants, 41.7% exhibited cognitive impairment. Overweight in childhood and late life was associated with cognitive impairment among diabetes patients (OR 2.63, 95% CI 1.52–4.55; OR 1.32, 95% CI 1.03–1.69). Diabetes patients with cognitive impairment were more likely to report a body weight decline and appetite reduction in the past three months (OR 4.18, 95% CI 2.61–6.71; OR 4.41, 95% CI 2.67–7.29). Higher BMI, weight loss, and appetite reduction were positively correlated with cognitive impairment. Given the risk of cognitive impairment, we suggest that body weight and BMI decline should be monitored in patients with diabetes.

## Introduction

With population aging, the number of age-related diseases, such as cognitive impairment, is increasing^1^. In China, more than 10% of older individuals experience cognitive impairment, ranging from mild deficits to dementia^2,3^. Previous studies have shown that type 2 diabetes is associated with a higher risk of cognitive decline^4,5^, and the increasing prevalence of diabetes worldwide may influence the future health care burden due to cognitive impairment^5,6^. A recent meta-analysis reported that prevalence of cognitive impairment in patients with type 2 diabetes was 45.0%^7^. Therefore it is important to identify risk factors for cognitive impairment in patients with type 2 diabetes.

Body weight, as a modifiable risk factor, is related to both diabetes and cognitive impairment^8,9^. In the general population, studies have reported different associations between body mass index (BMI) and the risk of dementia^10^. Some observational studies and meta-analyses have reported positive associations, with high BMI or obesity in midlife is associated with increasing risk of dementia^11–18^. While, inverse relationships that being overweight in late life increased risk of dementia were reported in studies among elderly^19–21^. For diabetes, a study in South Korea found that BMI after mid-life was associated with high dementia risk among patients with new-onset diabetes^22^. A cohort study reported that diabetes patients with greater body weight variability had a higher risk of all types of dementia^23^.

In longitudinal studies, weight loss has been observed as early as 12 years before the diagnosis of dementia^24^., cognitive decline may also happen decades before dementia diagnosis. However, the impact of weight status and weight loss on cognitive impairment risk among patients with type 2 diabetes is limited in literature. Furthermore, patients with type 2 diabetes undergo behavioral modification to increase fitness and reduce adiposity during disease control^25^. In this study, we aimed to evaluated the associations of cognitive impairment with BMI and weight changes in both the short term and throughout life using data from diabetes patients in a hospital setting.

## Methods

### Participants

Patients with type 2 diabetes aged 60 years and above admitted to the Department of Endocrinology at Penglai People's Hospital from October 2021 to May 2022 were recruited for this study. (Type 2 diabetes was diagnosed by either fasting plasma glucose ≥ 126 mg/dl, non-fasting plasma glucose ≥ 200 mg/d). Participated patients were registered at the hospital for long term disease control or newly diagnosed with type 2diabetes at Penglai People's Hospital during the survey. Individuals with an age at diabetes diagnosis < 30 years were excluded. We also excluded subjects with hearing impairment, or severe mental disorder, diagnosed dementia and those that refused to participate in the survey. The final sample size was 1355. Questionnaires were administered by trained doctors and nurses to collect demographic and lifestyle factors. The study protocols were approved by the Chinese Academy Medical Sciences (CAMS&PUMC-IEC-2021-022), and the research have been performed in accordance with the Declaration of Helsinki. All research was performed in accordance with relevant regulations, and all participants provided written informed consent.

### Cognition measurement

The Montreal Cognitive Assessment (MoCA) administered to participants by trained investigators. The MoCA is an assessment tool for rapid screening of cognitive dysfunction. It measures cognitive function in several domains: attention and concentration, executive functioning, memory, language, visuospatial skills, conception thinking, calculations, and orientation. The MoCA has good sensitivity and specificity for the detection of cognitive impairment among patients over 60 years of age^26^. The MoCA total score ranges from 0 to 30, with higher values indicating better cognitive function. If the subject has 12 years of education or fewer, a point is added to his/her total score. Cognitive impairment was defined as MoCA scores < 26 in this study.

### BMI and covariates

BMI was calculated using weight (in kg) divided by height squared (in meters). We categorized body weight status using standard recommended cutoffs of BMI: underweight (< 18.5 kg/m^2^), normal (18.5–25 kg/m^2^), overweight (25–30 kg/m^2^), and obesity (> 30 kg/m^2^) for all individuals in late life. We collected self-reported body weight (underweight, normal, or overweight) in midlife (at 40–60 years) and childhood (< 18 years). We didn’t further distinguish between overweight and obesity to avoid recall and classification bias among elderly individuals. We also collected data on weight and appetite changes in the months prior to the survey using the following questions: “During the past three months, did you notice any changes in your appetite, such as reduced or severely reduced appetite?” and “During the past year, did your notice any changes in your weight?” The responses options for latter question were no change (stable), increased weight, or decreased weight (by 1–3 kg or by more than 3 kg).

Self-reported demographic information, medical histories, and lifestyle factors were collected using questionnaires. Fasting blood glucose values were measured during the physical examination. Age at diabetes onset was self-reported by the participants and used to calculate diabetes durations. Alcohol consumption was classified as drinking beer or wine in the past 2 years. Persons who reported smoking in the past 2 years were classified as smoker. We categorized smoking and alcohol intake as “Yes” or “No” without any quantity threshold in this study. Marital status was categorized as single, married, divorced, widowed, or other. We divided participants into two age groups (60–69 years or >  = 70 years), and three education levels (primary education (primary school and under), secondary education (middle school or high school), and college or more (university or college graduate, and postgraduate)).

### Statistical analysis

All statistical analyses in this study were performed using Stata software (version 17.0). Descriptive analyses were conducted for demographic information, lifestyle factors, and diabetes status using Chi-square test. Analytic statistics were first performed using univariate logistic regression (Model 1); followed by a model adjusting for age, sex and education (Model 2); lastly, the model further accounted for diabetes duration and fasting blood glucose (Model 3). We examined the relationship between cognitive impairment and weight status in childhood, midlife, and late life. For late life analyses, we further divided the patients into early late life (60–70 years) and late late life (over 70 years) according to participants’ ages at the time of the interview. We combined body weight status at in late life, midlife and childhood for each participant, and to explore different combinations of weight status on cognitive impairment using logistic regression. At all life stages, the normal weight at all life stages (Normal_Normal_Normal group) was set as the reference to calculate the odds ratios (OR) for each combination. Associations of weight or appetite changes with cognitive impairment were investigated using the same method.

### Ethical approval

All study participants provided written informed consent. The study protocols and consent forms were approved by the Chinese Academy Medical Sciences. (CAMS&PUMC-IEC-2021-022).

## Results

A total of 1355 diabetes patients were included in the study, with mean age of 67.8 (± 5.1) years. After completing the MoCA, 565 participants (41.7%) were considered to exhibit cognitive impairment. The median score of MoCA after education adjustment was 25 (25^th^–75th: 23–28) in this study. Age, sex, and marital status were not associated with cognitive impairment among diabetes patients. Alcohol consumption and smoking status were significantly associated with cognitive impairment (P = 0.004 and P = 0.008, respectively). Patients with a longer diabetes duration had a higher risk of cognitive impairment (P < 0.001). We divided the participants into two groups based on fasting blood glucose concentrations; the prevalence of cognitive impairment in the worse glucose control group was higher than that in the better glucose control group (P = 0.011) (Table 1).

**Table 1:** Demographic variables, lifestyle factors, and diabetes conditions of the 1355 participants.

	N (%)	Cognitive impairment	%	*χ*^*2*^	*P* value
Total	1355 (100%)	565	41.7		
Age groups (years)				0.02	0.88
60 ~ 69	877 (64.7%)	367	41.8		
≥ 70	478 (35.3%)	198	41.4		
Sex				0.76	0.385
Male	739 (54.5%)	316	42.8		
Female	616 (45.5%)	249	40.4		
Education				133.07	< 0.001
Primary education	260 (19.2%)	27	10.4		
Secondary education	646 (47.7%)	332	51.4		
College or higher	449 (33.1%)	206	45.9		
Marital status				3.19	0.525
Single	8 (0.6%)	2	25.0		
Married	1292 (95.3)	536	41.5		
Separated	13 (1.0%)	6	46.2		
Divorced	15 (1.1%)	9	60.0		
Widowed	27 (2.0)	12	41.7		
Alcohol consumption*				8.19	0.004
No	1159 (85.5%)	465	40.1		
Yes	196 (14.5%)	100	51.0		
Smoking status*				7.11	0.008
No	1114 (82.2%)	446	40.0		
Yes	241 (17.8%)	119	49.4		
Diabetes duration (years)				28.1	< 0.001
0–4	374 (27.6%)	114	30.5		
5–9	407 (30.0%)	196	48.2		
≥ 10	574 (42.4%)	255	44.4		
Fasting blood glucose				6.49	0.011
< 7.0 mmol/L	318 (23.5)	113	35.5		
≥ 7.0 mmol/L	1037 (76.5)	452	43.6		

*Alcohol consumption was classified as drinking beer or wine in the past 2 years. Persons who reported smoking in the past 2 years were classified as smoker.

For the age-specific analyses, overweight in late life (60 years and older) was associated with a higher risk of cognitive impairment after adjusting for age, sex, and education (OR 1.38, 95% CI 1.08–1.76). After further accounting for diabetes duration and fasting blood glucose, overweight in late life (60 years and older) was still associated with higher risk of cognitive impairment (OR 1.32, 95% CI 1.03–1.69). We further separated participants into early late life (60–69 years) and late life (70 years and older), and found that the association between overweight and cognitive impairment was significant in the 70 + years group. Overweight/obesity in childhood was related to cognitive impairment among diabetes patients in the fully adjusted model (OR 2.63, 95% CI 1.52–4.55). In contrast, underweight in midlife was associated with cognitive impairment in late life (OR 1.73, 95% CI 1.01–2.95). (Table 2).

**Table 2:** Associations of BMI and self-reported body weight with cognitive impairment among diabetes patients.

BMI	N	Cognitive impairment	%	MOD1			MOD2			MOD3		
				OR	95% CI	P value	OR	95% CI	P value	OR	95% CI	P value
At late life*												
Underweight	15	6	40	1.02	0.36–2.91	0.968	1.21	0.38–3.65	0.768	1.25	0.40–3.91	0.698
Normal	585	231	39.5	1			1			1		
Overweight	628	274	43.6	1.19	0.94–1.49	0.144	1.38	1.08–1.76	0.011	1.32	1.03–1.69	0.03
Obesity	127	54	42.5	1.13	0.77–1.67	0.527	1.42	0.93–2.17	0.108	1.29	0.84–1.99	0.238
At 60–69 years												
Underweight	4	2	50	1.48	0.21–10.62	0.697	1.21	0.16–8.99	0.85	1.19	0.15–9.16	0.871
Normal	372	150	40.3	1			1			1		
Overweight	414	177	42.8	1.11	0.83–1.47	0.49	1.19	0.88–1.61	0.252	1.15	0.85–1.56	0.371
Obesity	87	38	43.7	1.15	0.72–1.84	0.576	1.55	0.92–2.60	0.1	1.41	0.83–2.39	0.208
At 70 years and over												
Underweight	11	4	36.4	0.93	0.26–3.28	0.912	1.44	0.36–5.79	0.612	1.48	0.37–6.04	0.581
Normal	213	81	38	1			1			1		
Overweight	214	97	45.3	1.35	0.92–1.99	0.127	1.89	1.23–2.90	0.004	1.82	1.17–2.80	0.007
Obesity	40	16	40	1.09	0.54–2.17	0.814	1.26	0.59–3.66	0.546	1.2	0.56–2.54	0.639
At childhood**												
Underweight	69	31	44.9	1.22	0.75–1.98	0.432	1.13	0.68–1.88	0.643	1.03	0.61–1.72	0.923
Normal	1218	489	40.2	1			1			1		
Overweight/obesity	68	45	66.2	2.92	1.74–4.88	< 0.001	2.94	1.71–5.07	< 0.001	2.63	1.52–4.55	0.001
At mid-life**												
Underweight	65	37	56.9	1.84	1.11–3.05	0.018	1.75	1.03–2.98	0.037	1.73	1.01–2.95	0.046
Normal	1124	470	41.8	1			1			1		
Overweight/ obesity	166	58	34.9	0.75	0.53–1.05	0.093	0.77	0.54–1.10	0.146	0.71	0.49–1.02	0.066

1 Univariate model.

2 Multivariate model (adjusted by age, sex, and education).

3 Multivariate model (adjusting by age, sex, education, diabetes duration, and fasting blood glucose).

*We categorized body weight status using standard recommended cutoffs of BMI: underweight (< 18.5 kg/m2), normal (18.5 to 25 kg/m2), overweight (25 to 30 kg/m2), and obesity (> 30 kg/m2) for all individuals in late life.

**We collected self-reported weight (underweight, normal, or overweight) in midlife (at 40–60 years) and childhood (< 18 years).

We combined weight statuses in childhood, midlife, and late life to explore the effect of lifetime BMI patterns on cognitive impairment among diabetes patients. Because the numbers of patients with certain subtypes were too small, some analyses were underpowered for examining these impacts; thus, some combinations were excluded from the analysis. Table 3 indicates a transition from underweight at mid-life to overweight at late life is related to cognitive impairment (OR:3.50, 95%CI 1.18–10.37). Being overweight at all life stages is also associated with cognitive impairment (OR:2.52, 95%CI 1.23–5.16). In Table 3, all statistical significances were appeared within late life overweight group. It indicates that overweight in late life played a central role in the associations between lifetime BMI and cognitive impairment in late life.

**Table 3:** Associations of body weight status at late life, mid-life, and childhood, and with cognitive impairment.

Late life-Mid life-childhood	N	MOD1			MOD2			MOD3		
		OR	95% CI	P Value	OR	95% CI	P Value	OR	95% CI	P Value
Normal-normal-normal	517	1								
Normal-underweight-underweight	29	0.82	0.24–2.83	0.75	0.67	0.18–2.40	0.538	0.62	0.17–2.24	0.469
Normal-overweight-overweight	39	1.67	0.55–5.04	0.363	1.52	0.48–4.76	0.473	1.29	0.41–4.06	0.658
Underweight-normal-normal	13	1.23	0.41–3.70	0.717	1.23	0.39–3.84	0.724	1.31	0.41–4.16	0.65
Overweight-normal-normal	594	1.04	0.82–1.33	0.746	1.11	0.86–1.42	0.426	1.06	0.82–1.36	0.659
Overweight-underweight-underweight	34	3.72	1.31–10.59	0.014	3.64	1.25–10.56	0.017	3.50	1.18–10.37	0.024
Overweight-overweight-overweight	127	2.75	1.38–5.50	0.004	2.83	1.38–5.75	0.004	2.52	1.23–5.16	0.011

1 Univariate model.

2 Multivariate model (adjusted by age, sex, and education).

3 Multivariate model (adjusting by age, sex, education, diabetes duration, and fasting blood glucose).

*For groups with too low a sample size, it has been removed from the table because the results may not be reliable.

Table 4 shows the association of weight and appetite changes in the past months with cognitive impairment. Compared with stable weight, weight declines in the past three months were associated with a higher risk of cognitive impairment. After adjustment for age, sex, education, diabetes duration, and fasting blood glucose, a weight decline of 1–3 kg was associated with increased risk of cognitive impairment (OR 4,50, 95% CI 2.82–7.17). Reduced appetite in the past three months was associated with an increased risk of cognitive impairment. Both (self-reported) reduced appetite and severely reduced appetite were associated with a high risk of cognitive impairment in the fully adjusted model (P < 0.05).

**Table 4:** Associations of weight changes and appetite reduction with cognitive impairment among diabetes patients.

	N	Cognitive impairment	%	MOD1			MOD2			MOD3		
				OR	95% CI	P Value	OR	95% CI	P value	OR	95% CI	P value
Weight change in the past year												
None (stable)	1208	463	38.3	1			1			1		
Increased	27	14	51.9	1.73	0.81–3.72	0.158	1.59	0.72–3.53	0.252	1.34	0.60–2.99	0.468
Decline of 1–3 kg	112	82	73.2	4.4	2.85–6.79	< 0.001	4.5	2.82–7.17	< 0.001	4.18	2.61–6.71	< 0.001
Decline of > 3 kg	8	6	75	4.83	0.97–24.02	0.054	5.39	0.93–31.14	0.06	4.84	0.85–27.40	0.075
Appetite change in past 3 months												
None (stable)	1230	476	38.7	1			1			1		
Reduced	101	74	73.3	4.34	2.75–6.85	< 0.001	4.86	2.96–7.97	< 0.001	4.41	2.67–7.29	< 0.001
Severely reduced	16	14	87.5	11.09	2.51–49.00	0.002	8.56	1.93–38.04	0.005	9.63	2.14–43.25	0.003

1 Univariate model.

2 Multivariate model (adjusted by age, sex, and education).

3 Multivariate model (adjusting by age, sex, education, diabetes duration, and fasting blood glucose).

## Discussion

We enrolled 1355 diabetes patients from Penglai People Hospital to assess the association of BMI and weight changes with cognitive impairment. Self-report of overweight in childhood and being overweight in late adulthood were associated with cognitive impairment among diabetes patients. Those diabetes patients with cognitive impairment were more likely to report a weight decline and appetite reduction in the past months.

Being overweight in late life increased the risk of cognitive impairment among diabetes patients in this study, regardless of diabetes duration and glucose control. This phenomenon was more apparent in late life (i.e. participants 70 years or older). A high BMI was associated with increased risk of cognitive impairment, consistent with the interpretation that adipose tissue increases the risk of vascular disorders by secreting biologically active hormonal compounds^27^. Being overweight or obese in childhood was also related to cognitive impairment in this study. Childhood obesity may increase the risk of early diabetes onset, which leads patients to be more likely to develop cognitive impairment in late life. Furthermore, obesity can cause the accumulation of brain lesions due to vascular and metabolic abnormalities, which may increase the risk of cognitive impairment^27^. Being underweight at midlife was found to be associated with cognitive impairment among diabetes patients in this study. This may be partly because some diabetes patients experienced emaciation before the onset of diabetes at midlife, at which point they might change lifestyles and accept treatment for disease control.

The associations between life span BMI and cognitive impairment among diabetes patients were not static, however, the underlying mechanism remains unclear and requires further in-depth investigation. We compared different combinations of BMI in childhood, mid-life and late life, as shown in Table 3, and found that being overweight at late life had the greatest influence. This suggests that elderly diabetes patients should maintain a healthy weight to decrease the risk of cognitive impairment.

Although being overweight at the time of survey was associated with cognitive impairment, patients with cognitive impairment in this study reported weight decline in the past year. Our previous studies also found that in the general population, BMI decline starting in late midlife was associated with an increased risk of dementia^8^. In this study, we focused on patients with diabetes, and explored the impact of body weight on the risk of cognitive impairment. Our results are consistent with previous findings regarding the effect of declining BMI on cognitive impairment. A nationwide cohort study in the US found that weight loss increased dementia risk among elderly inpatients, who were “expected to have more comorbidities and to have diabetes”^28^. A randomized intervention study in Finland reported significant cognitive declines with decreasing BMI among overweight patients accompanied by impaired glucose tolerance^29^. A Korean cohort reported that weight loss after diabetes onset was associated with higher risk of Alzheimer’s disease^22^. In our study, a weight loss of 1–3 kg was associated with cognitive impairment, but a weight loss of > 3 kg in the past months was not (Table 3). This might explain the contradiction between the findings that both overweight and weight loss in late life are associated with cognitive impairment. Although patients with cognitive impairment may have exhibited weight loss, the decline was not severed and didn’t change their overweight status.

The severity of diabetes and concurrent conditions may also be associated with cognitive impairment. We added the duration of diabetes and fasting blood glucose in model 3 as adjustments, taking into account the diabetic status of patients to some extent. Depression may have a negative impact on self-management through cognitive function. Pantiens with diabetes have a higher prevalence of depression compared to the general population. Therefore, interactions between diabetes and co-existing conditions should be considered in future studies. For the topic of BMI in this study, the pathophysiological pathways linking BMI change and cognitive impairment may be mediated by cardiovascular risk factors, lifestyle factors, or insulin resistance. Patients with incident diabetes are likely to maintain strict glycemic control and healthy lifestyle changes, which can lead to BMI declines^30^. Declines in weight might also occur during cognitive decline due to difficulty swallowing, loss of initiative, and loss of taste, which results in skipping meals or forgetting to eat. This was further supported by our finding that patients with cognitive impairment experienced appetite reduction in the past three months.

There are some limitations in this study. First, this study is cross-sectional in design and cannot establish a causal relationship between BMI and cognitive impairment. Although we retrospectively assessed participants' body weight at childhood and midlife via self-report, recall bias may exist, especially among elderly individuals. Participants with cognitive impairment, especially those affected by memory impairment, may not accurately recall their past body weight. Furthermore, for weight and appetite, we did not use an objective survey scale, but used a questionnaire to collect the self-reported weight and appetite of the elderly. This is because the elderly in this study had poor acceptance of the objective scale, we adopted a form that was more acceptable to the elderly for information collection. Such a collection method may lead to a bias in the results as well. Second, confounders such as physical activity and diet were not measured in this study or included in the models. HbA1c level- an common measurement of diabetes disease control used in other research, was not collected in the survey and thus was not evaluated in this study. Similarly, genes such as apolipoprotein E (APOE) are strongly associated with cognitive impairment and could have confounded the studied associations^31,32^. Third, diabetes treatment status was not adjusted for in the models because all participants in the hospital were undergoing drug treatment for diabetes during the investigations. However, information on antidiabetic drugs or their dosage was not available for this analysis. Fourth, the detection of cognitive impairment was based on MoCA scores; this instrument has been demonstrated to exhibit sensitivity and specificity for cognitive impairment. However, cognitive functioning should be assessed in greater detail by professional clinicians in future studies. Last, participants in this study were diabetes patients in a hospital setting; thus, it is possible that the reported associations may not be fully generalizable to other context.

In summary, the present study showed that being overweight at late life was associated with cognitive impairment among diabetes patients. Furthermore, weight loss and appetite reduction were positively correlated with cognitive impairment. Given the risk of cognitive impairment, we suggest that body weight and BMI decline should be monitored in patients with diabetes.

## Data Availability

The datasets generated and/or analyzed during the current study have been deposited in the National Population Health Data Center, https://www.ncmi.cn/. The accession of the data can be obtained by registering on the National Population Health Data Center and contacting the corresponding author.
